# Biological and Synthetic Surfactants Increase Class I Integron Prevalence in Ex Situ Biofilms

**DOI:** 10.3390/microorganisms12040712

**Published:** 2024-03-31

**Authors:** Ralf Lucassen, Nicole van Leuven, Dirk Bockmühl

**Affiliations:** Faculty of Life Sciences, Rhine-Waal University of Applied Sciences, Marie-Curie-Str. 1, 47533 Kleve, Germany; ralf.lucassen@hochschule-rhein-waal.de (R.L.); nicole.vanleuven@hochschule-rhein-waal.de (N.v.L.)

**Keywords:** class I integron, surfactant, resistance, anti-microbial, biofilm

## Abstract

The role of biocides in the spread of antimicrobial resistance (AMR) has been addressed but only a few studies focus on the impact of surfactants on microbial diversity and AMR, although they are common constituents of cleaners, disinfectants, and personal care products and are thus released into the environment in large quantities. In this study, we used a static ex situ biofilm model to examine the development of four biofilms exposed to surfactants and analyzed the biofilms for their prevalence of class I integrons as a proxy for the overall abundance of AMR in a sample. We furthermore determined the shift in bacterial community composition by high-resolution melt analysis and 16S ribosomal RNA (*16S rRNA*) gene sequencing. Depending on the initial intrinsic prevalence of class I integrons in the respective ex situ biofilm, benzalkonium chloride, alkylbenzene sulfonate, and cocamidopropyl betaine increased its prevalence by up to 6.5× on average. For fatty alcohol ethoxylate and the biosurfactants sophorolipid and rhamnolipid, the mean increase did not exceed 2.5-fold. Across all surfactants, the increase in class I integrons was accompanied by a shift in bacterial community composition. Especially benzalkonium chloride, cocamidopropyl betaine, and alkylbenzene sulfonate changed the communities, while fatty alcohol ethoxylate, sophorolipid, and rhamnolipid had a lower effect on the bacterial biofilm composition.

## 1. Introduction

One of the leading health threats of the 21st century is the ever-growing number of pathogenic bacteria resistant to a growing number of even last-resort antibiotics. It is estimated that 1.2 million deaths in 2019 were directly attributable to bacteria resistant to multiple antibiotics [1]. Misuse and overuse of antibiotics in healthcare settings, but also in livestock breeding, are considered to be the main drivers in the development of multidrug-resistant pathogens. However, the role of environmental factors such as anthropogenic pollution should not be underestimated. In this respect, biocides are frequently used in clinical, public, and private settings in disinfectants and special cleaners, as well as in personal care products. For QACs (quaternary ammonium compounds) like benzalkonium chloride (BKC) it was shown that sub-inhibitory concentrations can promote antibiotic resistance by co-resistance via class I integrons and that the transfer of this antimicrobial resistance can be plasmid-borne [2,3]. Class I integrons are of special interest, because they capture, integrate, and express gene cassettes under a common promoter. They confer several phenotypes including resistance to a broad range of antibiotic classes, heavy metals, and biocides. Environmental stress leads to the integration of new gene cassettes and thus the relative prevalence (RP) of the clinical version of the class I integron-integrase gene (*intI1*) per 16S rRNA gene copy is a proxy for anthropogenic pollution and the overall AMR of bacteria in a sample [3,4,5].

In the domestic setting, AMR has not been investigated comprehensively, yet there are some studies that found a high abundance of resistant bacteria in different household-related settings, such as washing machines and dishwashers [5]. These findings may at least partly be explained by the action of biocides, such as QACs or oxygen bleach, both of which are frequently used in automated dishwashing and laundering. However, some studies also suggest a high prevalence of AMR-related class I integrons in domestic areas where frequent use of biocidal actives must not be assumed, such as the shower drain [3]. It seems obvious that other substances that are being used on a regular basis might affect the level of antimicrobial resistance in these environments, with surfactants being a likely candidate. They are present in most cleaning and personal care products and thus have an almost permanent contact with bacterial biofilms in appliances and drains.

Planktonic bacterial cells initiate the formation of a biofilm through aggregation. These aggregated bacteria can adhere to a solid surface. Bacterial aggregates produce extracellular polymeric substrates (EPS) that can contain polysaccharides, proteins, lipids, and extracellular DNA (eDNA). These compounds maintain a rigid biofilm structure and promote the integrity and survival of the bacteria. Cell surface proteins, adhesins but also eDNA influence the adherence process [6] and are therefore targets of surfactants. Besides the general cell solubilization and permeabilization properties of surfactants, the interaction of surfactants with these compounds depends strongly on their structure, and effects can range from simple anti-aggregation and anti-adhesion to strong biocidal action. Cationic surfactants have strong antibacterial activity against a wide range of Gram-negative and Gram-positive bacteria. In particular, cationic surfactants with 10–14 carbon atoms in the chain show high biocidal efficacy. Anionic surfactants are strong solubilizers and have moderate antibacterial activity against Gram-positive but limited activity against Gram-negative bacteria. The antibacterial effects of nonionic surfactants increase by increasing hydrophobic chain length and are reduced by increasing ethoxylate chain length. Biosurfactants exhibit the same functionality as chemically synthetized surfactants, but biosurfactants are more environmentally friendly, with lower toxicity and higher biodegradable ability [7,8].

However, only a few studies focus on the impact of surfactants on microbial diversity and antimicrobial resistance gene (ARG) abundance, although they are major constituents of the earlier-mentioned products and are released into the environment in large quantities. There is evidence that surfactants contribute to the dissemination of AMR and/or lower species richness in contaminated environments [9,10,11]. Lower species richness can in turn, lead to a higher prevalence of resistant bacteria. This has been shown in a wastewater treatment plant receiving wastewater from bulk drug production and in the gut microbiome of humans and non-human primates [12,13]. Furthermore, sub-inhibitory concentrations of surfactants can permeabilize bacterial membranes and promote plasmid-borne ARGs horizontal gene transfer in *E. coli* DH5α [14]. In addition, permeabilization can induce ROS (reactive oxygen species) and DNA damage. Since class I integron integrase gene expression is activated by SOS response, after DNA damage, this could increase ARG transfer as well [15,16]. To further clarify the role of surfactants in the spread of AMR, we tested a range of synthetic and biologically produced surfactants for their potential to increase the prevalence of class I integrons, which serves as a proxy for the overall AMR in a sample. To this end, we have adapted a static ex situ biofilm model according to a protocol by Ledwoch et al. that allows for the investigation of these effects in a complex microbial community. Ex situ biofilm models have been used in similar approaches and were shown to provide a comprehensive, yet stable lab system for the investigation of biofilms [17]. In contrast to single-species biofilms, these systems not only represent a model that might react to environmental impacts, such as the treatment with antibiotics, biocides, or surfactants, in a more realistic manner, but also allow for the investigation of changes in the microbial community as such, which is a prerequisite for the current study.

Biofilms in this study were harvested from standard household siphons. These biofilms contain a large array of bacteria and, depending on the sampling location, a varying prevalence of *intI1*. Furthermore, this prevalence was shown to correlate well with the overall prevalence of multi-resistant bacteria in different environmental samples [3,5]. Ex situ biofilms were cultivated in a low-nutrient culture medium under the selective pressure of different surfactants. After cultivation and media exchange over a course of 7 days, the prevalence of *intI1* was determined by qPCR as previously described [3]. In addition, bacterial community composition was assessed by the use of HRMA (high-resolution melt analysis) and *16S rRNA* gene sequencing data [18].

## 2. Materials and Methods

### 2.1. Sampling of Biofilms

Biofilms were collected by swab method (Copan Diagnostics Inc., Murrieta, CA, USA) from standard household siphons (bathroom sink, kitchen sink, shower 1, and shower 2) in Kleve, Germany. The biofilm was transferred into a 50 mL reaction tube (Sarstedt AG & Co. KG., Nümbrecht, Germany) containing 25 mL of PBS solution (Carl Roth GmbH + Co. KG, Karlsruhe, Germany). After vigorous vortexing for 2 min, the suspension was mixed 1/1 with 80% glycerol. The biofilm suspension was homogenized by end-over-end rotation (IKA-Werke GmbH & Co. KG, Staufen, Germany) for 5 min. Additionally, 1 mL aliquots were prepared and stored at −80 °C.

### 2.2. Preparation of Culture Medium

For culturing of biofilms, a mixture of 2.5% tryptic soy broth (Merck Millipore, Darmstadt, Germany) and 2.5% malt extract broth (Merck Millipore, Darmstadt, Germany) was used. Tryptic soy broth and malt extract broth were prepared according to the manufacturer’s instructions. The resulting media (100%) were each mixed to 2.5% in sterilized tap water of 5.43°e (Clark degree), which corresponds to 0.77 mmol/L CaCO_3_. The final pH was 6.8.

### 2.3. Ex Situ Biofilm Model

For cultivation of ex situ biofilms, 50 mL of culture medium (mixture of 2.5% tryptic soy broth and 2.5% malt extract broth) was applied to a 50 mL test tube (Sarstedt AG & CO. KG., Nümbrecht, Germany). Following the addition of 1 mL of biofilm suspension and vortexing for 1 min, 1 mL was applied to each well of a 24-well cell culture plate (Sarstedt AG & CO. KG., Nümbrecht, Germany). Samples were incubated at 25 °C for 6 h without the addition of surfactants. After 6 h, the surfactants were applied by addition of 1 mL of the respective surfactant (at 2× of the desired concentration) in culture medium. Samples were incubated with regular exchange of culture medium (with surfactant) after 24 h of incubation over the course of 5 days. The medium was exchanged by tilting the plate and drawing the medium from the plate with a micropipette. A final incubation step of 48 h (without exchange of culture medium after 24 h) was performed before DNA extraction. Ex situ biofilms incubated in surfactant-free medium were processed in the same way and served as the control. All tests were conducted in independent triplicate determination.

### 2.4. Surfactants

Benzalkonium chloride (12060-100G) was purchased from Sigma Aldrich (St. Louis, MO, USA), cocamidopropyl betaine (Dehyton PK 45) and alkylbenzene sulfonate (Disponil LDBS 55) were purchased from BASF (Ludwigshafen, Germany), fatty alcohol ethoxylate (Marlipal 24/70) was purchased from Sasol (Johannesburg, South Africa), and rhamnolipid (Rewoferm RL 100) and sophorolipid (Rewoferm SL ONE) were purchased from Evonik (Essen, Germany). The surfactants were dissolved in culture medium (mixture of 2.5% tryptic soy broth and 2.5% malt extract broth). All surfactants were added to the biofilm model to a final concentration of 0.1% and 0.01% (*w*/*v*), except for benzalkonium chloride, which had to be applied at 0.01% and 0.001% (*w*/*v*) because of its higher biocidal activity. The critical micelle concentrations (CMC) of the surfactants are given in Table 1.

In general, the CMC depends on the composition of the solution with respect to salt concentration and pH, and the actual CMC in our culture medium may vary from the values in Table 1.

### 2.5. Extraction of DNA

After 7 days the culture medium was aspirated and discarded by use of a micropipette. DNA was extracted according to the instructions of FastDNA Spin Kit for Soil (MP Biomedicals Germany GmbH, Eschwege, Germany), with the exception that 978 µL of PBS (from Kit) and 122 µL of MT Buffer (from Kit) were added to the wells of the cell culture dish directly. After a 5 min incubation step at room temperature, the biofilm was detached by use of a cotton swap. The biofilm suspension was then transferred to the matrix tube of the extraction kit and extraction procedure was continued. The extracted DNA was quantified by Nanodrop (Thermo Fisher Scientific Inc., Waltham, MA, USA).

### 2.6. qPCR and High-Resolution Melt Analysis (HRMA)

For determination of *16S rRNA* and *intI1* genes, qPCR was performed on QuantStudio 3 (Applied Biosystems by Thermo Fisher Scientific Inc.). A 1 µL amount of purified sample DNA, standards, low prevalence and high prevalence controls, and non-template control (qPCR grade water) were applied to 10 µL of master mix. We used sewage sludge from a wastewater treatment plant (WWTP) and non-contaminated soil samples from organic farm fields (used for plant breeding; fertilized with organic manure only) as high and low prevalence controls. Amplicons of *intI1* and *16S rRNA* genes from Pseudomonas aeruginosa (isolated from WWTP) served as standards (10^3^ to 10^8^ copies/mL). Copies/mL of *intI1* and *16S rRNA* genes were determined in the same test run but in separate wells. All determinations were performed in duplicates.

The master mix consisted of 5 µL FastStart Essential DNA Green Master (Roche Life Sciences, Mannheim, Germany), 4.8 µL of PCR grade water, and 0.1 µL of 10 µM forward and reverse primer, respectively. qPCR was performed using the following parameters: 95 °C/10 min initial activation and denaturation followed by 33 cycles of 95 °C/15 sec denaturation, 60 °C/15 s annealing, and 72 °C/15 s extension. A final 72 °C/90 sec final extension step was included. Subsequently, a melting curve analysis from 50 °C to 95 °C at 0.02 °C/s was performed. In case of negative results, 1/10 and 1/100 dilutions of the sample were analyzed to avoid false negative results due to inhibitors.

For the determination of *intI1*, the primers F165 (5′CGAACGAGTGGCGGAGGGTG′3) and R476 (5′TACCCGAGAGCTTGGCACCCA′3) were used [4]. This primer pair targets the clinical version of class I integron integrase gene and has been proposed as a good marker for ARG and phenotypic resistance of a sample [3,22]. For the determination of *16S rRNA* gene, the primers F919 (5′GAATTGACGGGGGCCCGCACAAG′3) and R1378 (5′CGGTGTGTACAAGGCCCGGGAACG′3) were used [23]. For the HRMA ITS1f (5′-TTGTACACACCGCCCG-′3) and ITS2r (5′-YGCCAAGGCATCCACC-′3) primer set was employed [23]. The bacterial ITS primer set was used for HRMA instead of the *16S rRNA* gene primers because the ITS sequence offers a higher degree of variation and thus better discrimination of melting curves [24].

### 2.7. Determination of Relative Class I Integron Prevalence

Based on the qPCR result the relative prevalence (RP) in % of *intI1* was determined according to the following equation.

(1) $$RP=\left(\frac{copies\;per\;mL\;of\;intI1}{copies\;per\;ml\;of\;16S\;rRNA\;gene}\right)*2.5*100$$

Based on a previous study [3], we used an average value of 2.5 copies of the *16S rRNA* gene per bacterial cell, which is a matter for discussion because other studies use a value of four copies [25,26]. Although not influencing the data of this study, this should be considered when comparing studies that used different copy numbers for the *16S rRNA* gene.

### 2.8. Calculation of Euclidian Distance from HRMA as a Measure of Bacterial Community Dissimilarity

HRMA and calculation of Euclidian distance between to samples is a good proxy for the dissimilarity of microbial communities [13]. After qPCR and subsequent HRMA, data were normalized between y = 0 and y = 1 across the active melting range of 72–92 °C. Euclidian distances between treated (culture medium plus surfactant) and non-treated (culture medium only) biofilms were calculated according to the following formula.

(2) $$=\sqrt{{{(x}_{2}-x_{1})}^{2}+{{(y}_{2}-y_{1})}^{2}\;}$$

### 2.9. 16S rRNA Gene Sequencing and Determination of the Dissimilarity of Bacterial Communities

To verify the utility of HRMA analysis in determining the dissimilarity of the biofilms and to obtain a better understanding of the bacterial communities, the extracted DNA of the four biofilms (incubated at 0.1% of the respective surfactant and in case of benzalkonium chloride at 0.01%) was pooled and analyzed by *16S rRNA* gene sequencing.

The pooling of the same amount of DNA from four biofilms (treated with the same surfactant) and subsequent sequencing was completed to obtain an overview of the phylogenetic taxa and mainly to support the results of the HRMA (dissimilarity of treated samples and untreated control). This way, we obtained a mean result across the different biofilms and a trend of what we find in reality in siphons from different sources.

Sequencing was completed by the external service provider Eurofins Genomic (Ebersberg, Germany) on Illumina platform, using the V3 and V4 region of the *16S rRNA* gene, for bacterial identification. Reads were assigned to the taxonomic unit kingdom, phylum, class, order, family, genus, and species. Dissimilarity was calculated across all taxonomic units using unweighted UniFrac distance. We used the unweighted UniFranc distance in favor of weighted UniFrac distance because unweighted Unifrac considers only presence and absence information and counts the fraction of branch length unique to either community [27]. All taxonomic units with less than 0.1% of reads were collapsed in the category “Other”. If the representative sequence of an OTU had no significant database match at the respective taxonomic unit, the total number of reads of these unclassified OTUs is stated as category “Unclassified”. Sequencing data were uploaded and can be accessed via https://opus4.kobv.de/opus4-rhein-waal/frontdoor/index/index/docId/1871, uploaded on 15 March 2024.

### 2.10. Statistics

For calculations, data analysis, and preparation of graphs, GraphPad Prism 10.1.0 (GraphPad Software Inc., San Diego, CA, USA) and Microsoft Excel 2016 were used. Dunnett’s multiple comparison test was used to determine the significance of the variation between the treated and non-treated samples.

## 3. Results

### 3.1. Influence of Surfactants on intI1 Prevalence in a Developing Biofilm

**Figure 1:**
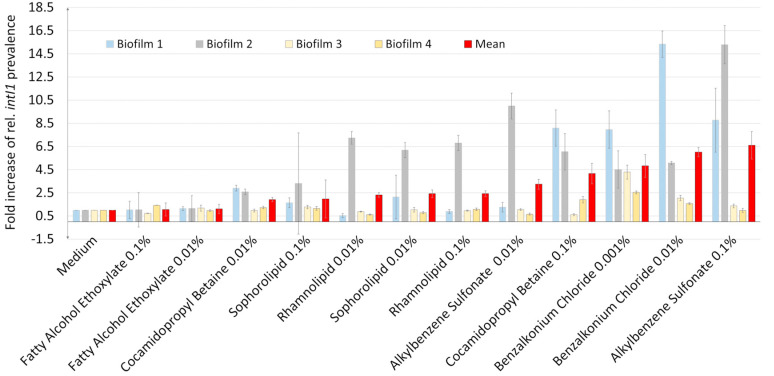
Fold increase in *intI1* prevalence and standard deviation: (Biofilm 1) Ex situ kitchen siphon biofilm (low intrinsic *intI1* prevalence of 0.45%). (Biofilm2) Ex situ bathroom siphon biofilm (medium intrinsic *intI1* prevalence of 2.45%). (Biofilm 3) Ex situ shower biofilm (high intrinsic *intI1* prevalence of 18.65%). (Biofilm 4) Ex situ shower biofilm (very high intrinsic *intI1* prevalence of 35.63%). All surfactants were added to the biofilm model to a final concentration of 0.1% and 0.01% (*w*/*v*), except for benzalkonium chloride, which had to be applied at 0.01% and 0.001% (*w*/*v*). Triplicate determinations were performed for each biofilm. Red bars indicate the mean value of all four biofilms.

Figure 1 shows the influence of surfactants on *intI1* prevalence in a developing biofilm. Depending on the intrinsic *intI1* prevalence (biofilm in culture medium; without surfactants), the ability of the surfactants to increase *intI1* prevalence varied strongly between biofilms. At a low intrinsic *intI1* prevalence (0.45), especially alkylbenzene sulfonate, cocamidopropyl betaine, and benzalkonium chloride increased *intI1* prevalence between eight- and sixteen-fold. In contrast, non-ionic surfactants (fatty alcohol ethoxylate, sophorolipid, and rhamnolipid) had little to no effect. At medium *intI1* prevalence (2.45), all surfactants increased *intI1* prevalence, except for the fatty alcohol ethoxylate.

At high (18.85) intrinsic *intI1* prevalence, only benzalkonium chloride increased *intI1* prevalence slightly by 4.5-fold. At a very high (35.63) intrinsic *intI1* prevalence, all surfactants had a rather low influence on *intI1* prevalence.

### 3.2. Correlation of the Fold Increase in intI1 Prevalence and Shift in Bacterial Community

To analyze the influence of the different surfactants on the prevalence of *intI1* in general and to see whether this increase is accompanied by a shift in the bacterial community, we correlated the mean rel. *intI1* prevalence values with the mean Euclidian distance of bacterial *ITS* melt curves.

**Figure 2:**
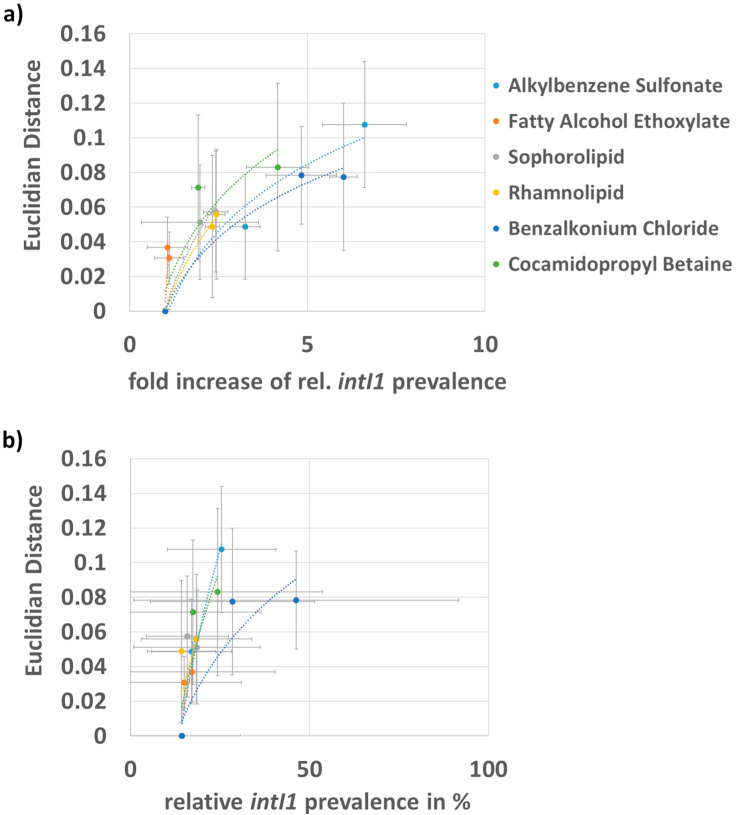
Mean fold increase in *intI1* and mean relative *intI1* prevalence blotted against the respective mean Euclidian distance of biofilms 1–4: (**a**) mean correlation of fold increase in *intI1* prevalence and Euclidian distance and (**b**) mean correlation of relative *intI1* prevalence and Euclidian distance. Biofilms were cultivated in absence and presence of different surfactants. All surfactants were added to the biofilm model to a final concentration of 0.1% and 0.01% (*w*/*v*), except for benzalkonium chloride, which had to be applied at 0.01% and 0.001% (*w*/*v*). Grey bars indicate the standard deviations.

The mean values reveal a clearer trend. However, since the effect of the surfactants on the biofilms was found to vary strongly between different sources (cp. Figure 2a), this might only be considered a general trend, which might not be applied to unknown biofilms. While the nonionic surfactant fatty alcohol ethoxylate had a weak effect on the bacterial composition and the fold increase in *intI1* prevalence, the nonionic bio surfactants sophorolipid and rhamnolipid had an intermediate effect on the bacterial composition but did increase *intI1* prevalence by 2–3-fold. In contrast, alkylbenzene sulfonate as an anionic surfactant had a strong effect on the bacterial composition and increased *intI1* prevalence up to 7–8-fold. The zwitterionic surfactant cocamidopropyl betaine increased *intI1* prevalence by 4–5-fold with a strong shift in bacterial community composition. Benzalkonium chloride, being a cationic surfactant with biocidal activity, increased *intI1* prevalence by up to six-fold and shifted the bacterial community strongly. With respect to the relative prevalence of *intI1*, these trends can be seen as well. Here, benzalkonium chloride had the strongest influence on relative *intI1* prevalence but it must be pointed out that the variation between the four biofilms was very high, as can be abstracted from the high standard deviation (Figure 2b). Dunnett’s multiple comparison test reveals that differences between samples are not significant. This indicates that, depending on the taxonomic constitution and intrinsic *intI1* prevalence, the biofilms are affected in very different ways by the same surfactant.

### 3.3. 16S rRNA Gene Sequencing Results

**Figure 3:**
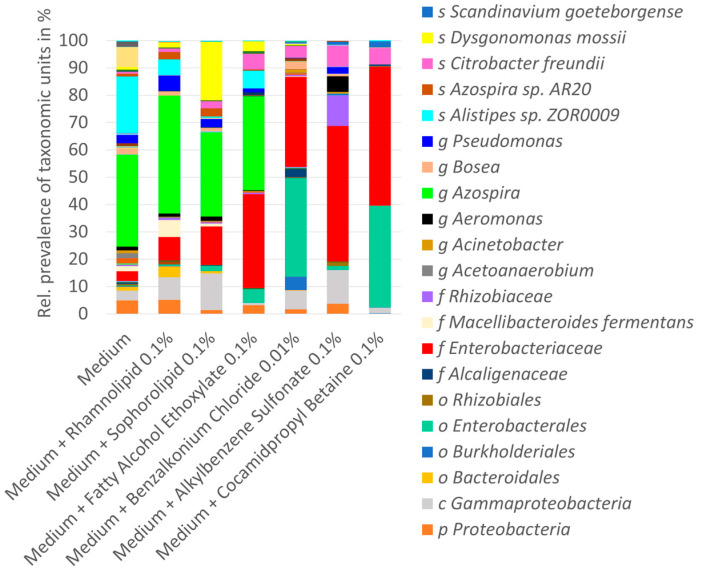
*16S rRNA* gene sequencing data from pooled DNA of four ex situ biofilms, cultured in the absence and presence of surfactants. The graphic contains the entire spectrum of identified phylogenetic taxa. However, for better overview, the legend contains only taxa with a rel. prevalence ≥ 0.5%. The letter in front of the respective taxonomic unit indicates s = species, g = genus, f = family, o = order, c = class, and *p* = phylum.

To verify the usability of HRMA analysis in the determination of bacterial dissimilarity of the biofilms and to receive a better understanding of the bacterial communities, extracted DNA of the four biofilms was pooled for some test conditions and subsequently sequenced. Figure 3 compiles the distribution of the respective bacterial taxa across the different treatments. *Enterobacteriaceae* represented the largest family group and dominated the bacterial communities. All surfactants increased their relative prevalence, with cocamidopropyl betaine showing the strongest influence. *Enterobacterales* was highly enriched by benzalkonium chloride and cocamidopropyl betaine. Furthermore, benzalkonium chloride, cocamidopropyl betaine, and alkylbenzene sulfonate reduced the genus *Azospira*, while *Alistipes* sp. was reduced by all surfactants. Across all treatments, *Citrobacter freundii* was the most abundant bacterial species, followed by *Scandinavium goeteborgense*. Interestingly, fatty alcohol ethoxylate, rhamnolipid, and especially sophorolipid enriched *Dysgonomonas mosii*.

*Alistepes* sp. and *Citrobacter freundii* are colonizers of the gastrointestinal tract and belong to the family of *Enterobacteriaceae*. Both species are associated with disease, form biofilms, and can carry resistance to multiple antibiotics [28,29].

*Scandinavium goeteborgense* and *Dysgonomonas mosii* are emerging pathogens. *Scandinavium goeteborgense* is a new member of the *Enterobacteriaceae* family isolated from a wound infection. The species can carry a novel quinolone resistance gene variant. *Dysgonomonas mosii* was isolated from infected blood, is known to form biofilms, and is also multi-resistant to a large number of antibiotics [30,31].

However, the generation of species data from *16S rRNA* gene sequencing data is challenging and did deliver a large number of unclassified reads (between 60% and 90%) at this phylogenetic level.

The shifts in bacterial community composition as observed in HRMA were also verified by the calculation of UniFrac distance (Table 2). By this means, it could be confirmed that alkylbenzene sulfonate and benzalkonium chloride shift the population the most, followed by cocamidopropyl betaine, sophorolipid, and rhamnolipid. Fatty alcohol ethoxylate changes the community only slightly. It must be pointed out that the HRMA analysis revealed a more pronounced increase in the Euclidian distance in the case of cocamidopropyl betaine compared to sophorolipid and rhamnolipid.

### 3.4. Correlation of CMC and intI1 Prevalence

**Figure 4:**
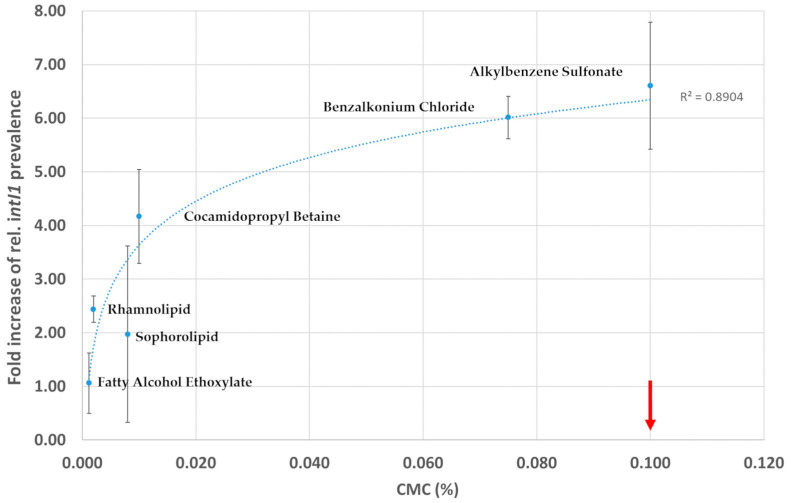
Correlation of fold increase in the relative prevalence class I integrons (*intI1*) to the critical micelle concentration (CMC) of the respective detergents. Error bars depict the standard deviation of *intI1* prevalence. The red arrow indicates the actual test concentration. Surfactants with a CMC close to this concentration have a stronger influence on class I integron prevalence.

A look at the CMC (see Table 1) of the respective surfactants can explain the very different influence of the surfactants on class I integron prevalence. Surfactants with a low CMC such as fatty alcohol ethoxylate, rhamnolipid, and sophorolipid have a lower impact on the prevalence of class I integrons. For surfactants with a higher CMC like cocamidopropyl betaine, alkylbenzene sulfonate, and benzalkonium chloride, we observed a strong increase in class I integron prevalence (Figure 4). For the test concentration of 0.1%, this means that surfactants with a CMC close to 0.1% were at least partially present as individual molecules or small aggregates. In contrast, surfactants with lower CMCs were mostly in the form of larger micelles.

## 4. Discussion

On average (across four ex situ biofilms), but with the exception of the nonionic surfactant fatty alcohol ethoxylate, all surfactants were found to increase the prevalence of class I integrons. Alkylbenzene sulfonate showed the strongest fold increase in *intI1*, followed by benzalkonium chloride and cocamidopropyl betaine. This increase was accompanied by a shift in the bacterial community as proven by HRMA and *16S rRNA* gene sequencing. We, therefore, assume that the increase in *intI1* prevalence may rather be caused by the selection of bacterial strains already harboring resistances than by horizontal gene transfer. However, the effect of a specific surfactant was found to be diverse depending on the initial intrinsic prevalence of *intI1* as well as (presumably) on the specific taxonomic constitution of the biofilm.

At a low to medium intrinsic prevalence (0.45–2.45) of *intI1*, the biofilms were more prone to an increase in *intI1* and a shift in the bacterial community by benzalkonium chloride, cocamidopropyl betaine, alkylbenzene sulfonate, sophorolipid, and rhamnolipid. At a higher intrinsic prevalence (18.65) of *intI1* the biofilm composition was only shifted strongly by the anionic surfactant alkylbenzene sulfonate and the zwitterionic surfactant cocamidopropyl betaine and cationic biocide benzalkonium chloride. Interestingly, for alkylbenzene sulfonate, this shift was not accompanied by an increase in *intI1* prevalence. At a very high intrinsic *intI1* prevalence, all surfactants shifted the bacterial population weakly with an increase in *intI1* by two-fold in the case of benzalkonium chloride. This points out that with a higher class I integron prevalence the bacterial communities seem to be more stable against stress factors like surfactants and only the cationic surfactant and strong biocide benzalkonium chloride has an effect on the already pre-selected community.

In particular, the strong effect of cocamidopropyl betaine on the proliferation of class I integrons and the enrichment of *Enterobacteriaceae* is of interest, as it is used in large quantities in personal care products such as shower gels and hair conditioners. This also explains the results of a previous study, where a very high prevalence of class I integrons was found especially in shower siphons [3]. Another finding of our study is the strong reduction in the nitrogen-fixing genus *Azospira* by the ionic surfactants and the enrichment of *Dysgonomonas mosii* by sub-inhibitory concentrations of fatty alcohol ethoxylate, rhamnolipid, and especially sophorolipid. This is of interest because Dysgonomonas mosii is an emerging opportunistic pathogen that can be multidrug resistant to even last-resort antibiotics [31].

The good correlation of the CMC of the respective surfactants, the increase in *intI1,* and the shift in the bacterial community might be explained by the fact that the tested concentrations of fatty alcohol ethoxylate, rhamnolipid, and sophorolipid are above the CMC and thus most of the surfactant molecules are bound in micelles. For cocamidopropyl betaine, alkylbenzene sulfonate, and benzalkonium chloride the tested concentration is close to the CMC and thus most of the surfactant is rather present as single molecules and not bound in micelles. This is important because the physicochemical and aggregation properties of surfactants allow individual surfactant molecules to migrate more efficiently through the peptidoglycan cell wall, for subsequent permeabilization of the inner membrane of Gram-negative and Gram-positive bacteria. In this respect, the peptidoglycan layer acts more as a filter through which the micelles are blocked and single molecules can pass more efficiently [32]. However, at some point, the concentration of single surfactant molecules or small aggregates is too low to permeabilize the inner membrane, although they can translocate through the cell wall.

Since only four exemplary biofilms with very different intrinsic *intI1* prevalences were used in this study, no statistical significance of the influence of the tested surfactants on the spread of bacterial resistance could be determined. Further studies to elucidate the precise role of surfactants in AMR using a larger but equally diverse set of well-characterized ex situ biofilms are mandatory. This could lead to the identification of compounds/products, which are drivers of AMR. In this respect, a non-static biofilm model like the drain biofilm model used by Ledwoch et al. might be favorable to resemble the life-like situation in more detail [12]. In such a study, surfactant concentrations above and below the CMC of a respective surfactant should be analyzed. However, since the CMC of a respective surfactant depends on the culture medium composition (e.g., salt content and pH value), the determination of the CMC under the given conditions should be part of such a study. It would also be favorable to know what surfactant concentrations are actually present in the environment (e.g., siphons) to have an even more life-like approach.

However, our study is the first to show that surfactants may be crucial for the development and dissemination of *intI1*-mediated AMR. Metagenomic analyses may be favorable to determine the degree of horizontal gene transfer vs. the increase in AMR by simple selection of the already resistant strains. By doing so, it may be possible to reveal surfactants with a low and high tendency to increase AMR as a recommendation for regulatory bodies and manufacturers.

## Figures and Tables

**Table 1:** The CMC of the surfactants, taken from the supplier’s technical data sheets (TDS) or, in the case of sophorolipid, rhamnolipid, and benzalkonium chloride, from published results.

Surfactant	CMC [g/L]	CMC [%]	Source
Fatty alcohol ethoxylate (Marlipal 24/70)	0.012	0.0012	TDS
Rhamnolipid (Rewoferm RL 100)	0.02	0.002	[19]
Sophorolipid (Rewoferm SL ONE)	0.08	0.008	[19]
Cocamidopropyl betaine (Dehyton PK 45)	0.1	0.01	TDS
Alkylbenzene sulfonate (Disponil LDBS 55)	1	0.1	TDS
Benzalkonium chloride	0.05–0.1	0.005–0.1	[20,21]

**Table 2:** Dissimilarity calculated by UniFrac distance (unweighted) of pooled DNA of four ex situ biofilms cultured in the absence and presence of surfactants in triplicate determination.

UniFrac Distance (Unweighted)	Mean
Medium + alkylbenzene sulfonate 0.1%	0.65
Medium + benzalkonium chloride 0.01%	0.51
Medium + cocamidopropyl betaine 0.1%	0.37
Medium + sophorolipid 0.1%	0.34
Medium + rhamnolipid 0.1%	0.33
Medium + fatty alcohol ethoxylate 0.1%	0.26
Medium	0.00

## Data Availability

*16S rRNA* gene sequencing data are available via https://opus4.kobv.de/opus4-rhein-waal/frontdoor/index/index/docId/1871, uploaded on 15 March 2024.
